# Systematic screening for skin, hair, and nail abnormalities in a large-scale knockout mouse program

**DOI:** 10.1371/journal.pone.0180682

**Published:** 2017-07-10

**Authors:** John P. Sundberg, Soheil S. Dadras, Kathleen A. Silva, Victoria E. Kennedy, Gaven Garland, Stephen A. Murray, Beth A. Sundberg, Paul N. Schofield, C. Herbert Pratt

**Affiliations:** 1 The Jackson Laboratory, Bar Harbor, Maine, United States of America; 2 Departments of Dermatology and Pathology, University of Connecticut, Farmington, Connecticut, United States of America; 3 Department of Physiology Development and Neuroscience, University of Cambridge, Cambridge, United Kingdom; Ludwig-Maximilians-Universitat Munchen, GERMANY

## Abstract

The International Knockout Mouse Consortium was formed in 2007 to inactivate (“knockout”) all protein-coding genes in the mouse genome in embryonic stem cells. Production and characterization of these mice, now underway, has generated and phenotyped 3,100 strains with knockout alleles. Skin and adnexa diseases are best defined at the gross clinical level and by histopathology. Representative retired breeders had skin collected from the back, abdomen, eyelids, muzzle, ears, tail, and lower limbs including the nails. To date, 169 novel mutant lines were reviewed and of these, only one was found to have a relatively minor sebaceous gland abnormality associated with follicular dystrophy. The B6N(Cg)-*Far2*^*tm2b(KOMP)Wtsi*^/2J strain, had lesions affecting sebaceous glands with what appeared to be a secondary follicular dystrophy. A second line, B6N(Cg)-*Ppp1r9b*^*tm1*.*1(KOMP)Vlcg*^/J, had follicular dystrophy limited to many but not all mystacial vibrissae in heterozygous but not homozygous mutant mice, suggesting that this was a nonspecific background lesion. We discuss potential reasons for the low frequency of skin and adnexal phenotypes in mice from this project in comparison to those seen in human Mendelian diseases, and suggest alternative approaches to identification of human disease-relevant models.

## Introduction

How many genes are involved in the normal ontogeny and physiology of the skin and its adnexal structures? How do they interact and how are they controlled? These questions, simple to articulate, have complex answers at best, but are predicated on our knowledge of gene function and more importantly the pleiotropic functions of genes, which can only be elucidated though experimentation and particularly through genetic manipulation. Faced with this challenge, we might initially search major databases such as Mouse Genome Informatics (MGI; http://www.informatics.jax.org/), Online Mendelian Inheritance in Man (https://www.omim.org/), or Orphanet (http://www.orpha.net/) and ask whether naturally-occurring or genetically engineered mutations show skin and skin-associated phenotypes; this can then be the beginning of a detailed investigation of gene expression, function and control. However, we only have experimentally-derived functional information for about half (11,973) of the protein-coding genes in the mouse as assessed by Gene Ontology annotation (MGI statistics; data accessed 19 Feb 2017) of the estimated 24,211 total, and while there are now many electronically inferred annotations for cellular location, process, and function for many more genes, these are not reliable predictors of the biological process in which genes may be involved. Critically, we have some (in many cases sparse) phenotype information from only 3,710 genes in Online Mendelian Inheritance in Man (accessed 19 Feb 2017). This is therefore greatly enhanced by gene/phenotype data from genetically variant mouse strains for around 9,493 mouse genes with human orthologs. The consequence of this is that although the mouse provides a great deal more functional information than the human, much of the genome remains biologically featureless, its “dark matter”, and it is only by mutating genes one by one that we can begin to build up a picture of their involvement in processes and structures, and their patterns of functional pleiotropy which are fundamentally unpredictable from sequence alone. An additional problem with mutant mice made in pursuit of hypothesis-driven science is that they are often made on highly variable or undefined genetic backgrounds, often for particular purposes, without systematic or standardized whole animal phenotyping, making it very difficult to compare phenotypes between mutant mice in any uniform way.

To address this problem on a large-scale basis, an international consortium was formed in 2007 to inactivate (“knockout”) all protein-coding genes in the mouse genome in embryonic stem cells using multiple gene targeting approaches on a uniform C57BL/6N genetic background [1, 2]. The second phase of this grand plan, the large-scale production and characterization of these mice, is now underway [3, 4]. The International Mouse Phenotyping Consortium was formed in 2011, composed of 17 Institutes across the world in 11 countries with 5 national funding agencies. This includes the NIH-supported contribution to these efforts, the Knockout Mouse Phenotyping Program, which consists of 3 groups; 1) The Jackson Laboratory, The 2) Baylor College of Medicine, The Wellcome Trust Sanger Institute and MRC Harwell Consortium, and 3) the University of California at Davis, The Toronto Center for Phenogenomics, Children’s Hospital Oakland Research Institute, and Charles River Laboratories Consortium). These consortia have generated over 5,000 strains of live mice using these embryonic stem cell lines and have recently transitioned to using CRISPR/Cas9-induced null alleles. Knockout lines are subjected to detailed, systematic evaluation (phenotyping). A majority of the phenotyping tests performed as part of these programs focus on physiological characterization as their primary goal [5]. While this approach generates an enormous amount of physiological data (clinical pathology, serology/hematology, behavioral data, etc.) [6, 7], histopathology, especially when done by highly trained experts focusing on specific organs, can often make the direct correlation quickly and accurately, as histologic evaluation by pathologists remains the definitive assay in human and veterinary clinical medicine for making most diagnoses [8, 9]. Furthermore, diseases of some organ systems are only truly defined at the gross clinical level followed by histopathology, special stains, and other tissue based tests, such as immunohistochemistry or electron microscopy. The skin, and its adnexa, is one such system.

While abnormalities of the skin and adnexa (eccrine glands, pilosebaceous unit, hair shaft, and nails) are easily seen by just looking at the mouse with the naked eye or with magnification (dissection or scanning electron microscope), detailed histopathology is still necessary to accurately define the abnormality in the skin and some lesions are only apparent microscopically. A primary high-throughput phenotyping strategy concentrates on wide coverage of a large number of parameters in a large number of mice and consequently is designed to pick up relatively clear-cut examples of phenodeviance.

Prior to the initiation of the current international efforts, both Lexicon and Deltagen undertook large-scale systematic mutagenesis and phenotyping projects, of which Lexicon’s used systematic histopathology as a routine phenotyping tool (see [10] for discussion) and generated histopathological analyses on nearly 5,000 mutant strains. Detailed descriptions of these strains are now in the process of being published in the literature [11, 12].

More recently, the Sanger Mouse Genetics Program reported a preliminary general histopathology workup of 50 randomly selected knockout lines yielding 2 with skin phenotypes, using single samples of dorsal skin from two males and two female mice [13]. This was complemented by a “special skin workup” as part of an extended primary phenotyping pipeline [14]. The histopathology protocol involved collecting one piece of dorsal skin, fixing, and processing it routinely to create hematoxylin and eosin stained slides. Nearly 800 mutant mouse skin samples were collected from 562 mutant lines in this high-throughput protocol. One and sometimes two slides with two or three serial sections of dorsal skin from one mouse in the study were provided. Mice were approximately 15 weeks of age. Only 23 lines (4.1%) had some sort of skin phenotype while all the others were normal, implying that many of the genes inactivated had no function in the skin; at least not evident from this screening protocol.

The International Mouse Phenotyping Consortium database currently (accessed 17 February 2017) shows that overall 2.22% of 3,199 lines show abnormalities of skin morphology and 5.35% of 2,971, abnormalities of the adnexa. This group reported no abnormalities of skin physiology in its database to date. A search of the Europhenome database, which overlaps with the International Mouse Phenotyping Consortium, shows 12 genes with annotations on skin morphology and physiology (excluding nociception; 27) of 563 genotypes derived from thesemutagenesis efforts (2.1%). The low number of skin phenotypes in these samples contrasts the frequent reporting of skin and adnexal phenotypes both in human Mendelian diseases and in the gathered data of MGI. Detailed examination of the MGI database shows that 1,069 of 9,045 targeted genes with phenotypes are annotated to either skin morphology or phenotypes; approximately 12%. In OMIM, 1,314/3,710 gene entries with phenotypic annotations are annotated to “abnormality of skin, HP:000095” or its children in the Human Phenotype Ontology database (35%) [15]. Assuming that we might expect skin and adnexal phenotypes in both humans and mice to result from loss of function of the same gene, the key question is where are the missing phenotypes? Even considering the exceptionally detailed phenotyping carried out in clinical practice, this is a large discrepancy. One possibility is that in the mouse, loss of function genes involved in the development or function of the skin and adnexa might be embryonic or neonatal lethal. However, this may also be an issue of detection; either through skewed phenotyping (i.e. not focused on the skin or not reported) or failure to detect phenotypes. There are projects associated with the US KnockOut Mouse Program that examine embryonic and perinatal lethality, yet none of them look beyond gross skin phenotype as reasons for early mortality [4]. From the reasoning above, we believe this problem can be addressed through careful targeted skin phenotyping.

The Jackson Laboratory is one of three sites in the United States generating knockout mutant mice. Histopathology is an optional parameter in the phenotyping pipeline, and thus is not performed and/or reported universally from individual centers. To better address finding skin lesions, representative retired breeder mice were received, histology was done on skin and nails from multiple sites, and slides were screened by a veterinary pathologist specializing in phenotyping skin mutants. Candidate models were subsequently reviewed by a physician pathologist specializing in dermatopathology. In so doing 169 novel mutant lines were reviewed and of those only one was found to have a relatively minor sebaceous gland abnormality.

## Results

Skin, hair follicles, and nail units of both sexes were screened for lesions in 169 novel mutant lines of knockout mice generated at JAX (Table 1). Of these 116 (68.6%) were homozygous and 61 (36.1%) were heterozygous mutant mice. Four lines had both heterozygous and homozygous mice screened (2.4%). Of the heterozygous mice, 62.3% were pre-wean lethal and 11.5% were sub-viable, which is defined as yielding fewer than half of the expected number of homozygotes from an intercross (<12.5%). For these strains no homozygous mutant mice were evaluated. A total of 743 (2.2 average per line and sex) mice were necropsied and the skin and adnexa were evaluated histologically. Nine lines (two females and three males for heterozygotes only and three females and one male for homozygotes only) had only one sex evaluated.

**Table 1:** KOMP lines evaluated. Clinical summary of 169 KOMP strains screened for skin, hair, and nail abnormalities.

No.	Strain	JR #	Mutant Allele	No. Mice	Age (days)	Genotype	Skin	Hair Follicle	Seba-ceous Gland	Nail	Prenatal Lethality	Sub-viable
1	B6N(Cg)	24041	*4933427D14Rik*^*tm1*.*1(KOMP)Vlcg*^/J	2	138/161	HET						
	B6N(Cg)	24041	*4933427D14Rik*^*tm1*.*1(KOMP)Vlcg*^/J	2	138/161	HET						
2	B6N(Cg)	24902	*Acsf2*^*tm1*.*1(KOMP)Vlcg*^/J	1	218	HOM						
	B6N(Cg)	24902	*Acsf2*^*tm1*.*1(KOMP)Vlcg*^/J	3	218/222	HOM						
3	C57BL/6NJ	25814	*Adad1*^*em1J*^/J	2	272/325	HET						X
	C57BL/6NJ	25814	*Adad1*^*em1J*^/J	2	308/337	HET						X
4	C57BL/6NJ	25475	*Adad2*^*em1J*^/J	2	193/199	HET						
	C57BL/6NJ	25475	*Adad2*^*em1J*^/J	2	196/199	HET						
5	B6N(Cg)	18592	*Adora2b*^*tm1*.*1(KOMP)Vlcg*^/J	2	282	HOM						
	B6N(Cg)	18592	*Adora2b*^*tm1*.*1(KOMP)Vlcg*^/J	2	165/223	HET						
6	B6N(Cg)	18561	*Ahrr*^*tm1b(KOMP)Wtsi*^/J	2	41/138	HOM						
	B6N(Cg)	18561	*Ahrr*^*tm1b(KOMP)Wtsi*^/J	2	41/138	HOM						
7	B6N(Cg)	25117	*Akip1*^*tm1*.*1(KOMP)Vlcg*^/J	2	183 /189	HOM						
	B6N(Cg)	25117	*Akip1*^*tm1*.*1(KOMP)Vlcg*^/J	2	183/189	HOM						
8	B6N(Cg)	24334	*Akr1b8*^*tm1*.*1(KOMP)Vlcg*^/J	2	125	HOM						
	B6N(Cg)	24334	*Akr1b8*^*tm1*.*1(KOMP)Vlcg*^/J	2	125	HOM						
9	B6N(Cg)	24656	*Anapc15*^*tm1*.*1(KOMP)Vlcg*^/J	2	125/139	HET					X	
	B6N(Cg)	24656	*Anapc15*^*tm1*.*1(KOMP)Vlcg*^/J	2	166	HET					X	
10	B6N(Cg)	21510	*Ap4e1*^*tm1b(KOMP)Wtsi*^/J	2	266	HOM						
	B6N(Cg)	21510	*Ap4e1*^*tm1b(KOMP)Wtsi*^/J	2	198/359	HOM						
11	B6N(Cg)	22304	*Arhgef10*^*tm1*.*1(KOMP)Vlcg*^/J	1	95	HOM						
	B6N(Cg)	22304	*Arhgef10*^*tm1*.*1(KOMP)Vlcg*^/J	3	95/99	HOM						
12	B6N(Cg)	18607	*Arrdc1*^*tm1*.*1(KOMP)Vlcg*^/J	3	180/213	HET						
	B6N(Cg)	18607	*Arrdc1*^*tm1*.*1(KOMP)Vlcg*^/J	1	180	HET						
13	B6N(Cg)	18657	*Arsk*^*tm1b(KOMP)Wtsi*^/J	2	254	HOM						
	B6N(Cg)	18657	*Arsk*^*tm1b(KOMP)Wtsi*^/J	1	254	HOM						
14	B6N(Cg)	18660	*Asb10*^*tm2b(KOMP)Wtsi*^/J	2	173	HOM						
	B6N(Cg)	18660	*Asb10*^*tm2b(KOMP)Wtsi*^/J	2	120	HOM						
15	B6N(Cg)	22101	*Asf1a*^*tm1b(KOMP)Wtsi*^/2J	2	320/385	HET					X	
	B6N(Cg)	22101	*Asf1a*^*tm1b(KOMP)Wtsi*^/2J	2	362/386	HET					X	
16	B6N(Cg)	22305	*Atrip*^*tm1b(KOMP)Wtsi*^/J	2	340	HET					X	
	B6N(Cg)	22305	*Atrip*^*tm1b(KOMP)Wtsi*^/J	2	346	HET					X	
17	B6N(Cg)	24979	*Bbs7*^*tm1b(EUCOMM)Wtsi*^/J	2	191/192	HET						X
	B6N(Cg)	24979	*Bbs7*^*tm1b(EUCOMM)Wtsi*^/J	2	177/192	HET						X
18	B6N(Cg)	21751	*Btg2*^*tm1b(KOMP)Mbp*^/2J	2	223	HOM						
	B6N(Cg)	21751	*Btg2*^*tm1b(KOMP)Mbp*^/2J	2	204/352	HOM						
19	B6N(Cg)	18653	*Bzw2*^*tm1b(KOMP)Mpb*^/J	2	186	HOM						
	B6N(Cg)	18653	*Bzw2*^*tm1b(KOMP)Mpb*^/J	2	226	HOM						
20	B6N(Cg)	22307	*C1qa*^*tm1b(EUCOMM)Wtsi*^/3J	2	308	HOM						
	B6N(Cg)	22307	*C1qa*^*tm1b(EUCOMM)Wtsi*^/3J	2	308	HOM						
21	B6N(Cg)	18562	*C1qtnf5*^*tm1*.*1(KOMP)Vlcg*^/J	2	380/472	HOM						
	B6N(Cg)	18562	*C1qtnf5*^*tm1*.*1(KOMP)Vlcg*^/J	2	443	HOM						
22	B6N(Cg)	22098	*C3*^*tm1*.*1(KOMP)Vlcg*^/J	2	128/219	HOM						
	B6N(Cg)	22098	*C3*^*tm1*.*1(KOMP)Vlcg*^/J	2	128/225	HOM						
23	B6N(Cg)	21282	*C9*^*tm1(KOMP)Vlcg*^/J	1	198/206	HET						
	B6N(Cg)	21282	*C9*^*tm1(KOMP)Vlcg*^/J	2	269	HET						
24	B6N(Cg)	22418	*Cacna2d3*^*tm1b(KOMP)Wtsi*^/J	2	239	HOM						
	B6N(Cg)	22418	*Cacna2d3*^*tm1b(KOMP)Wtsi*^/J	2	239	HOM						
25	B6N(Cg)	23393	*Cast*^*tm1*.*1(KOMP)Vlcg*^/2J	2	111/171	HOM						
	B6N(Cg)	23393	*Cast*^*tm1*.*1(KOMP)Vlcg*^/2J	2	111/171	HOM						
26	B6N(Cg)	18111	*Ccd20*^*tm1(KOMP)Vlcg*^/J	2	337/339	HET					X	
27	B6N(Cg)	24349	*Ccl26*^*tm1*.*1(KOMP)Vlcg*^/J	3	216/232/ 251	HOM						
	B6N(Cg)	24349	*Ccl26*^*tm1*.*1(KOMP)Vlcg*^/J	1	157	HOM						
28	B6N(Cg)	22016	*Cd33*^*tm1*.*1(KOMP)Vlcg*^/J	4	147/321	HOM						
	B6N(Cg)	22016	*Cd33*^*tm1*.*1(KOMP)Vlcg*^/J	4	176/321	HOM						
29	B6N(Cg)	22335	*Ceacam16*^*tm1*.*1(KOMP)Wtsi*^/J	4	100/207/ 227/285	HOM						
	B6N(Cg)	22335	*Ceacam16*^*tm1*.*1(KOMP)Wtsi*^/J	4	100/207/ 273/285	HOM						
30	B6N(Cg)	18836	*Cenpo*^*tm1b(KOMP)Mbp*^/J	2	313/379	HET					X	
	B6N(Cg)	18836	*Cenpo*^*tm1b(KOMP)Mbp*^/J	2	354/379	HET					X	
31	B6N(Cg)	22819	*Cers5*^*tm2b(KOMP)Mbp*^/J	2	206	HOM						
	B6N(Cg)	22819	*Cers5*^*tm2b(KOMP)Mbp*^/J	2	206	HOM						
32	B6N(Cg)	18603	*Chn1*^*tm1*.*1(KOMP)Vlcg*^/J	2	244/247	HOM						
	B6N(Cg)	18603	*Chn1*^*tm1*.*1(KOMP)Vlcg*^/J	2	244/246	HOM						
33	B6N(Cg)	25118	*Chp2*^*tm1b(KOMP)Mbp*^/J	3	149/210/ 211	HET						
	B6N(Cg)	25118	*Chp2*^*tm1b(KOMP)Mbp*^/J	2	210/211	HET						
34	B6N(Cg)	23317	*Chtf18*^*tm1b(KOMP)Wtsi*^/J	2	300	HET						X
	B6N(Cg)	23317	*Chtf18*^*tm1b(KOMP)Wtsi*^/J	2	300	HET						X
35	B6N(Cg)	18631	*Cinp*^*tm1*.*1(KOMP)Vlcg*^/J	2	126/136	HET					X	
	B6N(Cg)	18631	*Cinp*^*tm1*.*1(KOMP)Vlcg*^/J	4	131/135/ 136	HET					X	
36	B6N(Cg)	18587	*Cited4*^*tm1*.*1(KOMP)Vlcg*^/J	2	140/183	HOM						
	B6N(Cg)	18587	*Cited4*^*tm1*.*1(KOMP)Vlcg*^/J	2	140/183	HOM						
37	B6N(Cg)	23666	*Cldn19*^*tm1*.*1(KOMP)Vlcg*^/J	2	230/324	HOM						
	B6N(Cg)	23666	*Cldn19*^*tm1*.*1(KOMP)Vlcg*^/J	2	230/255	HOM						
38a	B6N(Cg)	18604	*Col18a1*^*tm1*.*1(KOMP)Vlcg*^/J	2	193/208	HET						
	B6N(Cg)	18604	*Col18a1*^*tm1*.*1(KOMP)Vlcg*^/J	2	191/208	HET						
38b	B6N(Cg)	18604	*Col18a1*^*tm1*.*1(KOMP)Vlcg*^/J	2	322/350	HOM						
	B6N(Cg)	18604	*Col18a1*^*tm1*.*1(KOMP)Vlcg*^/J	2	322/350	HOM						
39	B6N(Cg)	21485	*Cp*^*tm1b(KOMP)Wtsi*^/J	2	184/326	HOM						
	B6N(Cg)	21485	*Cp*^*tm1b(KOMP)Wtsi*^/J	2	271/326	HOM						
40	B6N(Cg)	22015	*Crym*^*tm1b(KOMP)Wtsi*^/J	2	161/198	HOM						
	B6N(Cg)	22015	*Crym*^*tm1b(KOMP)Wtsi*^/J	2	161/198	HOM						
41	B6N(Cg)	22096	*Cyb5d1*^*tm1*.*1(KOMP)Vlcg*^/J	3	323/354	HOM						
	B6N(Cg)	22096	*Cyb5d1*^*tm1*.*1(KOMP)Vlcg*^/J	1	355	HOM						
42	B6N(Cg)	24035	*Cyb5d2*^*tm1*.*1(KOMP)Wtsi*^/J	2	203	HOM						
	B6N(Cg)	24035	*Cyb5d2*^*tm1*.*1(KOMP)Wtsi*^/J	2	203	HOM						
43	B6N(Cg)	22777	*Dbf4*^*tm1b(KOMP)Wtsi*^/J	2	137/155	HET					X	
	B6N(Cg)	22777	*Dbf4*^*tm1b(KOMP)Wtsi*^/J	2	178	HET					X	
44	B6N(Cg)	27202	*Dbn1*^*tm1a(KOMP)Wtsi*^/J	2	274	HET						X
	B6N(Cg)	27202	*Dbn1*^*tm1a(KOMP)Wtsi*^/J	2	200/241	HET						X
45	B6N(Cg)	23604	*Dcps*^*tm1b(EUCOMM)Hmgu*^/J	2	249	HET					X	
	B6N(Cg)	23604	*Dcps*^*tm1b(EUCOMM)Hmgu*^/J	2	258	HET					X	
46	B6N(Cg)	18632	*Dnajb3*^*tm1*.*1(KOMP)Vlcg*^/J	2	209/240	HOM						
	B6N(Cg)	18632	*Dnajb3*^*tm1*.*1(KOMP)Vlcg*^/J	2	202/240	HOM						
47	B6N(Cg)	18580	*Dnajb7*^*tm1*.*1(KOMP)Vlcg*^/J	1	250	HOM						
	B6N(Cg)	18580	*Dnajb7*^*tm1*.*1(KOMP)Vlcg*^/J	2	268	HOM						
48	B6N(Cg)	18597	*Dnajb12*^*tm1*.*1(KOMP)Vlcg*^/J	2	156/243	HOM						
49	B6N(Cg)	18616	*Dnajc5g*^*tm1*.*1(KOMP)Vlcg*^/J	2	301	HOM						
	B6N(Cg)	18616	*Dnajc5g*^*tm1*.*1(KOMP)Vlcg*^/J	2	164	HOM						
50	B6N(Cg)	18637	*Dnajc14*^*tm1*.*1(KOMP)Vlcg*^/J	4	136/177	HOM						
	B6N(Cg)	18637	*Dnajc14*^*tm1*.*1(KOMP)Vlcg*^/J	4	117/177/ 352	HOM						
51	B6N(Cg)	18633	*Dnajc16*^*tm1*.*1(KOMP)Vlcg*^/J	1	122	HET						X
	B6N(Cg)	18633	*Dnajc16*^*tm1*.*1(KOMP)Vlcg*^/J	2	122	HET						X
52	B6N(Cg)	21508	*Dnase1l2*^*tm1*.*1(KOMP)Wtsi*^/J	2	326	HOM						
53	B6N(Cg)	26437	*Enox1*^*tm1*.*1(KOMP)Vlcg*^/J	3	239	HET						
	B6N(Cg)	26437	*Enox1*^*tm1*.*1(KOMP)Vlcg*^/J	1	266	HET						
54	B6N(Cg)	23489	*Ermp1*^*tm1*.*1(KOMP)Vlcg*^/J	4	167/201/ 220	HOM						
	B6N(Cg)	23489	*Ermp1*^*tm1*.*1(KOMP)Vlcg*^/J	4	170/173/ 201	HOM						
55	B6N(Cg)	24660	*Espnl*^*tm1b(KOMP)Wtsi*^/2J	2	180	HOM						
	B6N(Cg)	24660	*Espnl*^*tm1b(KOMP)Wtsi*^/2J	2	145	HOM						
56	C57BL/6NJ	25733	*Exoc4*^*em1J*^/J	4	121/123	HET					X	
	C57BL/6NJ	25733	*Exoc4*^*em1J*^/J	2	121/123	HET					X	
57	B6N(Cg)	18622	*Exosc8*^*tm1*.*1(KOMP)Vlcg*^/J	2	379	HET					X	
	B6N(Cg)	18622	*Exosc8*^*tm1*.*1(KOMP)Vlcg*^/J	2	429	HET					X	
58	B6N(Cg)	18705	*Fam161a*^*tm1b(KOMP)Wtsi*^/2J	2	209	HOM						
	B6N(Cg)	18705	*Fam161a*^*tm1b(KOMP)Wtsi*^/2J	2	253/316	HOM						
59	B6N(Cg)	22011	*Far2*^*tm2b(KOMP)Wtsi*^/2J	2	184	HOM		dystrophy	hypo-plasia			
	B6N(Cg)	22011	*Far2*^*tm2b(KOMP)Wtsi*^/2J	2	172/184	HOM		dystrophy	hypo-plasia			
60	B6N(Cg)	22097	*Foxo3*^*tm1*.*1(KOMP)Vlcg*^/J	2	148	HOM						
	B6N(Cg)	22097	*Foxo3*^*tm1*.*1(KOMP)Vlcg*^/J	2	226	HOM						
61	B6N(Cg)	21486	*Ghr*^*tm1b(KOMP)Wtsi*^/3J	2	304/337	HET						
	B6N(Cg)	21486	*Ghr*^*tm1b(KOMP)Wtsi*^/3J	2	304/337	HET						
62	B6N(Cg)	19459	*Ghrhr*^*tm1*.*1(KOMP)Vlcg*^/2J	1	292	HOM						
	B6N(Cg)	19459	*Ghrhr*^*tm1*.*1(KOMP)Vlcg*^/2J	2	141	HOM						
63	B6N(Cg)	18595	*Ghsr*^*tm1*.*1(KOMP)Vlcg*^/J	4	143/153/ 370	HOM						
	B6N(Cg)	18595	*Ghsr*^*tm1*.*1(KOMP)Vlcg*^/J	4	156/370	HOM						
64	B6N(Cg)	18643	*Gipc3*^*tm1b(KOMP)Wtsi*^/J	2	208	HOM						
	B6N(Cg)	18643	*Gipc3*^*tm1b(KOMP)Wtsi*^/J	2	208	HOM						
65	B6N(Cg)	18558	*Glycam1*^*tm1*.*1(KOMP)Vlcg*^/J	1	180	HOM						
	B6N(Cg)	18558	*Glycam1*^*tm1*.*1(KOMP)Vlcg*^/J	1	180	HOM						
66	B6N(Cg)	24058	*Gnb5*^*tm1*.*1(KOMP)Vlcg*^/J	2	174/185	HET						X
	B6N(Cg)	24058	*Gnb5*^*tm1*.*1(KOMP)Vlcg*^/J	2	186	HET						X
67	B6N(Cg)	18594	*H1fx*^*tm1*.*1(KOMP)Vlcg*^/J	2	189	HOM						
	B6N(Cg)	18594	*H1fx*^*tm1*.*1(KOMP)Vlcg*^/J	2	186/195	HOM						
68	C57BL/6NJ	25319	*Heyl*^*tm1b(KOMP)Wtsi*^/J	2	215	HOM						
	C57BL/6NJ	25319	*Heyl*^*tm1b(KOMP)Wtsi*^/J	2	215	HOM						
69	B6N(Cg)	24947	*Hfe2*^*tm1b(KOMP)Wtsi*^/J	2	168/197	HOM						
	B6N(Cg)	24947	*Hfe2*^*tm1b(KOMP)Wtsi*^/J	2	197/198	HOM						
70	B6N(Cg)	24981	*Hsf2*^*tm1b(KOMP)Wtsi*^/3J	2	172	HOM						
	B6N(Cg)	24981	*Hsf2*^*tm1b(KOMP)Wtsi*^/3J	2	172	HOM						
71	B6N(Cg)	22782	*Hspb1*^*tm1(KOMP)Vlcg*^/2J	2	188	HET						
72	B6N(Cg)	18619	*Hspb2*^*tm1*.*1(KOMP)Vlcg*^/J	4	262/263/ 386	HOM						
	B6N(Cg)	18619	*Hspb2*^*tm1*.*1(KOMP)Vlcg*^/J	4	262/263/ 386	HOM						
73	B6N(Cg)	18618	*Hspb3*^*tm1*.*1(KOMP)Vlcg*^/J	2	144/199	HOM						
	B6N(Cg)	18618	*Hspb3*^*tm1*.*1(KOMP)Vlcg*^/J	2	144/199	HOM						
74	B6N(Cg)	18617	*Htr1a*^*tm1*.*1(KOMP)Vlcg*^/J	2	345	HOM						
	B6N(Cg)	18617	*Htr1a*^*tm1*.*1(KOMP)Vlcg*^/J	2	137/308	HOM						
75	B6N(Cg)	24347	*Htr1d*^*tm1*.*1(KOMP)Vlcg*^/2J	2	165	HOM						
	B6N(Cg)	24347	*Htr1d*^*tm1*.*1(KOMP)Vlcg*^/2J	2	165/226	HOM						
76	B6N(Cg)	18615	*Htr3b*^*tm1*.*1(KOMP)Vlcg*^/J	2	299	HOM						
	B6N(Cg)	18615	*Htr3b*^*tm1*.*1(KOMP)Vlcg*^/J	2	299	HOM						
77	B6N(Cg)	23606	*Ift88*^*tm1*.*1(KOMP)Vlcg*^/J	2	350	HET					X	
	B6N(Cg)	23606	*Ift88*^*tm1*.*1(KOMP)Vlcg*^/J	2	349/350	HET					X	
78a	B6N(Cg)	21516	*Igsf11*^*tm1b(KOMP)Wtsi*^/J	1	297	HET						
	B6N(Cg)	21516	*Igsf11*^*tm1b(KOMP)Wtsi*^/J	3	287/300	HET						
78b	B6N(Cg)	21516	*Igsf11*^*tm1b(KOMP)Wtsi*^/J	2	280/351	HOM						
	B6N(Cg)	21516	*Igsf11*^*tm1b(KOMP)Wtsi*^/J	2	280	HOM						
79	B6N(Cg)	22303	*Il12rb2*^*tm1*.*1(KOMP)Vlcg*^/J	4	168/206/ 264	HOM						
	B6N(Cg)	22303	*Il12rb2*^*tm1*.*1(KOMP)Vlcg*^/J	4	168/206	HOM						
80	B6N(Cg)	22005	*Il24*^*tm1*.*1(KOMP)Vlcg*^/2J	4	64/165/194/214	HOM						
	B6N(Cg)	22005	*Il24*^*tm1*.*1(KOMP)Vlcg*^/2J	4	64/165/194/214	HOM						
81	B6N(Cg)	22388	*Il6st*^*tm1b(KOMP)Mbp*^/J	2	180/210	HOM						
	B6N(Cg)	22388	*Il6st*^*tm1b(KOMP)Mbp*^/J	2	210	HOM						
82	B6N(Cg)	18655	*Irf8*^*tm1b(KOMP)Wtsi*^/J	2	195	HOM						
	B6N(Cg)	18655	*Irf8*^*tm1b(KOMP)Wtsi*^/J	2	171/195	HOM						
83	B6N(Cg)	24055	*Jam2*^*tm1*.*1(KOMP)Mbp*^/J	2	140	HOM						
	B6N(Cg)	24055	*Jam2*^*tm1*.*1(KOMP)Mbp*^/J	2	140	HOM						
84	B6N(Cg)	26066	*Jmjd8*^*tm1*.*1(KOMP)Vlcg*^/J	3	269	HOM						
	B6N(Cg)	26066	*Jmjd8*^*tm1*.*1(KOMP)Vlcg*^/J	1	269	HOM						
85	B6N(Cg)	22821	*Jup*^*tm1*.*1(KOMP)Vlcg*^/J	2	109/143	HET					X	
	B6N(Cg)	22821	*Jup*^*tm1*.*1(KOMP)Vlcg*^/J	2	107/143	HET					X	
86	B6N(Cg)	21140	*Kcnh3*^*tm1*.*1(KOMP)Vlcg*^/2J	1	186	HOM						
	B6N(Cg)	21140	*Kcnh3*^*tm1*.*1(KOMP)Vlcg*^/2J	2	186/288	HOM						
87	B6N(Cg)	23603	*Kif1b*^*tm1b(KOMP)Wtsi*^/2J	2	319	HET					X	
	B6N(Cg)	23603	*Kif1b*^*tm1b(KOMP)Wtsi*^/2J	2	322	HET					X	
88	B6N(Cg)	22085	*Kif26b*^*tm2b(KOMP)Wtsi*^/J	2	323/446	HET					X	
	B6N(Cg)	22085	*Kif26b*^*tm2b(KOMP)Wtsi*^/J	2	327/446	HET					X	
89	B6N(Cg)	25381	*Klk14*^*tm1*.*1(KOMP)Vlcg*^/J	2	115/161	HOM						
	B6N(Cg)	25381	*Klk14*^*tm1*.*1(KOMP)Vlcg*^/J	2	115/152	HOM						
90	B6N(Cg)	23318	*Krt17*^*tm1*.*1(KOMP)Vlcg*^/2J	2	297/304	HET						
	B6N(Cg)	23318	*Krt17*^*tm1*.*1(KOMP)Vlcg*^/2J	2	297/304	HET						
91	B6N(Cg)	25383	*Krt9*^*tm1*.*1(KOMP)Vlcg*^/J	2	210	HOM						
	B6N(Cg)	25383	*Krt9*^*tm1*.*1(KOMP)Vlcg*^/J	2	210	HOM						
92	B6N(Cg)	25518	*Lipn*^*tm1*.*1(KOMP)Vlcg*^/J	4	188/189/ 195	HOM						
93	B6N(Cg)	18563	*Loxl1*^*tm1*.*1(KOMP)Vlcg*^/J	4	112/133/ 147	HET						
	B6N(Cg)	18563	*Loxl1*^*tm1*.*1(KOMP)Vlcg*^/J	2	110	HET						
94	B6N(Cg)	22094	*Lpar6*^*tm1*.*1(KOMP)Vlcg*^/J	2	172/226	HOM						
	B6N(Cg)	22094	*Lpar6*^*tm1*.*1(KOMP)Vlcg*^/J	2	172/213	HOM						
95	B6N(Cg)	23021	*Lrrc15*^*tm1b(KOMP)Wtsi*^/J	1	183	HOM						
	B6N(Cg)	23021	*Lrrc15*^*tm1b(KOMP)Wtsi*^/J	1	48	HOM						
96	B6N(Cg)	23024	*Mdk*^*tm1*.*1(KOMP)Mbp*^/J	2	193	HOM						
	B6N(Cg)	23024	*Mdk*^*tm1*.*1(KOMP)Mbp*^/J	2	193	HOM						
97a	B6N(Cg)	22092	*Mmp8*^*tm1(KOMP)Vlcg*^/J	2	213	HET						
97b	B6N(Cg)	22092	*Mmp8*^*tm1*.*1(KOMP)Vlcg*^/J	2	232/273	HOM						
	B6N(Cg)	22092	*Mmp8*^*tm1*.*1(KOMP)Vlcg*^/J	2	245/273	HOM						
98	B6N(Cg)	18635	*Mrps25*^*tm1*.*1(KOMP)Vlcg*^/J	2	303	HET					X	
	B6N(Cg)	18635	*Mrps25*^*tm1*.*1(KOMP)Vlcg*^/J	2	303/364	HET					X	
99	B6N(Cg)	24673	*Myo7b*^*tm1b(EUCOMM)Hmgu*^/J	2	237/240	HOM						
	B6N(Cg)	24673	*Myo7b*^*tm1b(EUCOMM)Hmgu*^/J	2	236/237	HOM						
100	B6N(Cg)	23599	*Nat1*^*tm1*.*1(KOMP)Vlcg*^/J	2	142/152	HOM						
	B6N(Cg)	23599	*Nat1*^*tm1*.*1(KOMP)Vlcg*^/J	2	142/157	HOM						
101	B6N(Cg)	18575	*Ncald*^*tm1*.*1(KOMP)Vlcg*^/J	3	230/315	HOM						
	B6N(Cg)	18575	*Ncald*^*tm1*.*1(KOMP)Vlcg*^/J	3	230/310/ 408	HOM						
102	B6N(Cg)	18577	*Nefh*^*tm1*.*1(KOMP)Vlcg*^/J	1	230	HOM						
	B6N(Cg)	18577	*Nefh*^*tm1*.*1(KOMP)Vlcg*^/J	1	230	HOM						
103	B6N(Cg)	18697	*Nemf*^*tm1b(EUCOMM)Hmgu*^/J	2	78	HET					X	
	B6N(Cg)	18697	*Nemf*^*tm1b(EUCOMM)Hmgu*^/J	2	78	HET					X	
104	B6N(Cg)	24185	*Nes*^*tm1b(KOMP)Wtsi*^/J	2	139/140	HOM						
	B6N(Cg)	24185	*Nes*^*tm1b(KOMP)Wtsi*^/J	2	162/170	HOM						
105	B6N(Cg)	18638	*Nmrk2*^*tm1*.*1(KOMP)Vlcg*^/J	2	172/177	HOM						
	B6N(Cg)	18638	*Nmrk2*^*tm1*.*1(KOMP)Vlcg*^/J	2	177/180	HOM						
106	B6N(Cg)	18645	*Nrcam*^*tm2e*.*1(KOMP)Wtsi*^/J	2	242/275	HOM						
	B6N(Cg)	18645	*Nrcam*^*tm2e*.*1(KOMP)Wtsi*^/J	2	242/275	HOM						
107	B6N(Cg)	24899	*Nsun7*^*tm1*.*1(KOMP)Vlcg*^/J	3	183/190	HET						
	B6N(Cg)	24899	*Nsun7*^*tm1*.*1(KOMP)Vlcg*^/J	1	183	HET						
108	B6N(Cg)	24233	*Nxn*^*tm1b(EUCOMM)Wtsi*^/J	2	232/236	HET					X	
	B6N(Cg)	24233	*Nxn*^*tm1b(EUCOMM)Wtsi*^/J	2	236	HET					X	
109	B6N(Cg)	24036	*Orc1*^*tm1b(KOMP)Wtsi*^/J	2	185	HET					X	
	B6N(Cg)	24036	*Orc1*^*tm1b(KOMP)Wtsi*^/J	2	185/206	HET					X	
110	B6N(Cg)	22338	*Osm*^*tm1b(KOMP)Wtsi*^/J	4	198/270/ 358/376	HOM						
	B6N(Cg)	22338	*Osm*^*tm1b(KOMP)Wtsi*^/J	4	198/270/ 358	HOM						
111	B6N(Cg)	23058	*Parp8*^*tm1*.*1(KOMP)Wtsi*^/J	2	202	HOM						
	B6N(Cg)	23058	*Parp8*^*tm1*.*1(KOMP)Wtsi*^/J	2	202	HOM						
112	B6N(Cg)	21613	*Parp16*^*tm1*.*1(KOMP)Mbp*^/2J	2	288	HOM						
	B6N(Cg)	21613	*Parp16*^*tm1*.*1(KOMP)Mbp*^/2J	2	265	HOM						
113	B6N(Cg)	22341	*Pcsk5*^*tm1b(KOMP)Mbp*^/J	2	306/321	HET					X	
	B6N(Cg)	22341	*Pcsk5*^*tm1b(KOMP)Mbp*^/J	2	346	HET					X	
114	B6N(Cg)	24330	*Pnmt*^*tm1*.*1(KOMP)Vlcg*^/J	4	120/140/ 152/182	HOM						
	B6N(Cg)	24330	*Pnmt*^*tm1*.*1(KOMP)Vlcg*^/J	3	140/182	HOM						
115a	B6N(Cg)	18609	*Ppp1r9b*^*tm1*.*1(KOMP)Vlcg*^/J	4	139/282	HET		vibrissa				
	B6N(Cg)	18609	*Ppp1r9b*^*tm1*.*1(KOMP)Vlcg*^/J	2	139/330	HET		vibrissa				
115b	B6N(Cg)	18609	*Ppp1r9b*^*tm1*.*1(KOMP)Vlcg*^/J	2	321	HOM						
	B6N(Cg)	18609	*Ppp1r9b*^*tm1*.*1(KOMP)Vlcg*^/J	2	114/137	HOM						
116	B6N(Cg)	22099	*Prep*^*tm1b(KOMP)Wtsi*^/J	2	328/350	HET					X	
	B6N(Cg)	22099	*Prep*^*tm1b(KOMP)Wtsi*^/J	2	350	HET					X	
117a	B6N(Cg)	18576	*Prom2*^*tm1*.*1(KOMP)Vlcg*^/J	3	89	HET						
	B6N(Cg)	18576	*Prom2*^*tm1*.*1(KOMP)Vlcg*^/J	1	89	HET						
117b	B6N(Cg)	18576	*Prom2*^*tm1*.*1(KOMP)Vlcg*^/J	2	403	HOM						
	B6N(Cg)	18576	*Prom2*^*tm1*.*1(KOMP)Vlcg*^/J	2	437	HOM						
118	B6N(Cg)	24025	*Psen1*^*tm1*.*1(KOMP)Vlcg*^/J	2	225/240	HET					X	
	B6N(Cg)	24025	*Psen1*^*tm1*.*1(KOMP)Vlcg*^/J	2	240	HET					X	
119	B6N(Cg)	23667	*Pycr1*^*tm1*.*1(KOMP)Vlcg*^/J	2	165/251	HOM						
	B6N(Cg)	23667	*Pycr1*^*tm1*.*1(KOMP)Vlcg*^/J	2	165/251	HOM						
120	B6N(Cg)	22387	*Rab5a*^*tm1b(KOMP)Wtsi*^/J	4	222/230	HOM						
	B6N(Cg)	22387	*Rab5a*^*tm1b(KOMP)Wtsi*^/J	4	222/227/ 230	HOM						
121	B6N(Cg)	22389	*Rab24*^*tm1(KOMP)Wtsi*^/J	2	179	HET						
	B6N(Cg)	22389	*Rab24*^*tm1(KOMP)Wtsi*^/J	2	243	HET						
122	B6N(Cg)	23422	*Rab27b*^*tm1b(KOMP)Wtsi*^/J	2	127/129	HOM						
	B6N(Cg)	23422	*Rab27b*^*tm1b(KOMP)Wtsi*^/J	2	133/159	HOM						
123	B6N(Cg)	24188	*Rab34*^*tm1b(EUCOMM)Hmgu*^/J	2	328	HET					X	
	B6N(Cg)	24188	*Rab34*^*tm1b(EUCOMM)Hmgu*^/J	2	328	HET					X	
124	B6N(Cg)	23664	*Rab36*^*tm1b(KOMP)Wtsi*^/J	2	96/255	HOM						
	B6N(Cg)	23664	*Rab36*^*tm1b(KOMP)Wtsi*^/J	2	96/255	HOM						
125	B6N(Cg)	23059	*Rab43*^*tm1*.*1(KOMP)Vlcg*^/J	2	130	HOM						
	B6N(Cg)	23059	*Rab43*^*tm1*.*1(KOMP)Vlcg*^/J	2	130	HOM						
126	B6N(Cg)	26882	*Rabac1*^*tm1*.*1(KOMP)Vlcg*^/J	2	118	HOM						
	B6N(Cg)	26882	*Rabac1*^*tm1*.*1(KOMP)Vlcg*^/J	2	118	HOM						
127	B6N(Cg)	25849	*Rbpj*^*tm1b(EUCOMM)Hmgu*^/J	2	298	HET					X	
	B6N(Cg)	25849	*Rbpj*^*tm1b(EUCOMM)Hmgu*^/J	2	255	HET					X	
128	B6N(Cg)	18572	*Resp18*^*tm1*.*1(KOMP)Vlcg*^/J	1	231	HOM						
	B6N(Cg)	18572	*Resp18*^*tm1*.*1(KOMP)Vlcg*^/J	2	231	HOM						
129	B6N(Cg)	24674	*Rhbdl2*^*tm1b(KOMP)Wtsi*^/J	2	138	HOM						
	B6N(Cg)	24674	*Rhbdl2*^*tm1b(KOMP)Wtsi*^/J	2	138	HOM						
130	B6N(Cg)	21750	*Rilpl2*^*tm1b(KOMP)Wtsi*^/J	4	150/198/ 287	HOM						
	B6N(Cg)	21750	*Rilpl2*^*tm1b(KOMP)Wtsi*^/J	4	242/287/ 295	HOM						
131	B6N(Cg)	21511	*Rnf10*^*tm1b(KOMP)Wtsi*^/J	2	222	HOM						
	B6N(Cg)	21511	*Rnf10*^*tm1b(KOMP)Wtsi*^/J	2	228	HOM						
132	B6N(Cg)	26493	*Rps6kl1*^*tm1*.*1(KOMP)Vlcg*^/J	2	242	HOM						
	B6N(Cg)	26493	*Rsp6kl1*^*tm1*.*1(KOMP)Vlcg*^/J	2	242	HOM						
133	B6N(Cg)	23136	*Sdha*^*tm2b(KOMP)Wtsi*^/2J	2	343/393	HET					X	
	B6N(Cg)	23136	*Sdha*^*tm2b(KOMP)Wtsi*^/2J	2	393/396	HET					X	
134	B6N(Cg)	24038	*Sgta*^*tm1b(KOMP)Mbp*^/J	2	250/254	HET						
	B6N(Cg)	24038	*Sgta*^*tm1b(KOMP)Mbp*^/J	2	250	HET						
135	B6N(Cg)	21142	*Sh3tc2*^*tm1b(KOMP)Wtsi*^/J	2	221	HOM						
	B6N(Cg)	21142	*Sh3tc2*^*tm1b(KOMP)Wtsi*^/J	1	227	HOM						
136	B6N(Cg)	23511	*Ska2*^*tm1b(KOMP)Wtsi*^/J	2	228	HET					X	
	B6N(Cg)	23511	*Ska2*^*tm1b(KOMP)Wtsi*^/J	2	231	HET					X	
137	B6N(Cg)	23414	*Slc25a35*^*tm1*.*1(KOMP)Vlcg*^/J	2	179	HOM						
	B6N(Cg)	23414	*Slc25a35*^*tm1*.*1(KOMP)Vlcg*^/J	2	179	HOM						
138a	B6N(Cg)	22780	*Sorbs2*^*tm1(KOMP)Mbp*^/J	1	86	HET						
	B6N(Cg)	22780	*Sorbs2*^*tm1(KOMP)Mbp*^/J	3	86/199	HET						
138b	B6N(Cg)	22780	*Sorbs2*^*tm1*.*1(KOMP)Mbp*^/J	1	159	HOM						
	B6N(Cg)	22780	*Sorbs2*^*tm1*.*1(KOMP)Mbp*^/J	2	159/160	HOM						
139	B6N(Cg)	23020	*Sox18*^*tm1*.*1(KOMP)Vlcg*^/J	2	178/226	HOM						
	B6N(Cg)	23020	*Sox18*^*tm1*.*1(KOMP)Vlcg*^/J	2	161/165	HOM						
140	B6N(Cg)	26095	*Spag4*^*tm1*.*1(KOMP)Mbp*^/J	2	161	HOM						
	B6N(Cg)	26095	*Spag4*^*tm1*.*1(KOMP)Mbp*^/J	1	59	HOM						
141	B6N(Cg)	18820	*Sptssa*^*tm1b(KOMP)Wtsi*^/J	2	287/292	HET					X	
	B6N(Cg)	18820	*Sptssa*^*tm1b(KOMP)Wtsi*^/J	2	327	HET					X	
142	B6N(Cg)	24184	*Srd5a3*^*tm1b(EUCOMM)Wtsi*^/J	2	258/265	HET					X	
	B6N(Cg)	24184	*Srd5a3*^*tm1b(EUCOMM)Wtsi*^/J	2	265	HET					X	
143	B6N(Cg)	21115	*Stag3*^*tm1e*.*1(KOMP)Wtsi*^*/2*J	1	234	HOM						
	B6N(Cg)	21115	*Stag3*^*tm1e*.*1(KOMP)Wtsi*^/2J	2	104/275	HOM						
144	B6N(Cg)	18611	*Stk16*^*tm1*.*1(KOMP)Vlcg*^/J	2	105	HET					X	
	B6N(Cg)	18611	*Stk16*^*tm1*.*1(KOMP)Vlcg*^/J	2	66/105	HET					X	
145	B6N(Cg)	18611	*Stk16*^*tm1*.*1(KOMP)Vlcg*^/J	2	39	HOM						
	B6N(Cg)	18611	*Stk16*^*tm1*.*1(KOMP)Vlcg*^/J	2	39	HOM						
146	B6N(Cg)	22781	*Stx16*^*tm1b(KOMP)Wtsi*^/J	1	301	HOM						
	B6N(Cg)	22781	*Stx16*^*tm1b(KOMP)Wtsi*^/J	2	301/320	HOM						
147	B6N(Cg)	22784	*Sycp3*^*tm1(KOMP)Vlcg*^/J	2	110	HET						
	B6N(Cg)	22784	*Sycp3*^*tm1(KOMP)Vlcg*^/J	2	183	HET						
148	B6N(Cg)	22784	*Sycp3*^*tm1*.*1(KOMP)Vlcg*^/J	4	197/214/ 274/279	HOM						
	B6N(Cg)	22784	*Sycp3*^*tm1*.*1(KOMP)Vlcg*^/J	4	165/202/ 274	HOM						
149	B6N(Cg)	23665	*Syn3*^*tm1*.*1(KOMP)Vlcg*^/J	2	267/270	HOM						
	B6N(Cg)	23665	*Syn3*^*tm1*.*1(KOMP)Vlcg*^/J	2	267/270	HOM						
150	B6.129S1	24694	*Tgm2*^*tm1Rmgr*^/J	2	230/234	HOM						
	B6.129S1	24694	*Tgm2*^*tm1Rmgr*^/J	2	203/234	HOM						
151	B6N(Cg)	24406	*Timm22*^*tm1b(KOMP)Wtsi*^/J	2	181/192	HET					X	
	B6N(Cg)	24406	*Timm22*^*tm1b(KOMP)Wtsi*^/J	2	193	HET					X	
152	B6N(Cg)	18639	*Timp3*^*tm1*.*1(KOMP)Vlcg*^/J	3	210/278/ 292	HOM						
	B6N(Cg)	18639	*Timp3*^*tm1*.*1(KOMP)Vlcg*^/J	1	210	HOM						
153	B6N(Cg)	18629	*Tmem136*^*tm1*.*1(KOMP)Vlcg*^/J	2	254/274	HOM						
	B6N(Cg)	18629	*Tmem136*^*tm1*.*1(KOMP)Vlcg*^/J	2	187/274	HOM						
154	B6N(Cg)	23060	*Tmem151b*^*tm1b(KOMP)Wtsi*^/J	4	219/221/ 288	HOM						
	B6N(Cg)	23060	*Tmem151b*^*tm1b(KOMP)Wtsi*^/J	4	202/288	HOM						
155	B6N(Cg)	26542	*Tpcn1*^*tm1*.*1(KOMP)Vlcg*^/J	3	174	HOM						
	B6N(Cg)	26542	*Tpcn1*^*tm1*.*1(KOMP)Vlcg*^/J	1	174	HOM						
156	B6N(Cg)	21752	*Tpgs2*^*tm1*.*1(KOMP)Vlcg*^/J	2	156	HOM						
	B6N(Cg)	21752	*Tpgs2*^*tm1*.*1(KOMP)Vlcg*^/J	2	156	HOM						
157	B6N(Cg)	18606	*Tprn*^*tm1*.*1(KOMP)Vlcg*^/J	2	270	HOM						
	B6N(Cg)	18606	*Tprn*^*tm1*.*1(KOMP)Vlcg*^/J	2	141/293	HOM						
158	B6N(Cg)	18553	*Trip13*^*tm1*.*1(KOMP)Vlcg*^/J	4	181/196/ 239	HOM						
	B6N(Cg)	18533	*Trip13*^*tm1*.*1(KOMP)Vlcg*^/J	4	41/181/321	HOM						
159	B6N(Cg)	21483	*Tspan18*^*tm1*.*1(KOMP)Vlcg*^/J	2	263	HOM						
	B6N(Cg)	21483	*Tspan18*^*tm1*.*1(KOMP)Vlcg*^/J	2	263	HOM						
160	C57BL/6NJ	26909	*Ttll10*^*em1J*^/J	2	200/229	HOM						
	C57BL/6NJ	26909	*Ttll10*^*em1J*^/J	2	196/229	HOM						
161	B6N(Cg)	22817	*Ube2c*^*tm1*.*1(KOMP)Wtsi*^/J	2	249/270	HET					X	
	B6N(Cg)	22817	*Ube2c*^*tm1*.*1(KOMP)Wtsi*^/J	2	287	HET					X	
162	B6N(Cg)	24027	*Ube2m*^*tm1*.*1(KOMP)Wtsi*^/2J	2	484	HET					X	
	B6N(Cg)	24027	*Ube2m*^*tm1*.*1(KOMP)Wtsi*^/2J	2	484	HET					X	
163	B6N(Cg)	23019	*Vsig8*^*tm1*.*1(KOMP)Mbp*^/J	1	116	HOM						
	B6N(Cg)	23019	*Vsig8*^*tm1*.*1(KOMP)Mbp*^/J	1	44	HOM						
164	B6N(Cg)	23660	*Zbtb4*^*tm1*.*1(KOMP)Vlcg*^/J	2	267	HOM						
	B6N(Cg)	23660	*Zbtb4*^*tm1*.*1(KOMP)Vlcg*^/J	2	144/267	HOM						
165	B6N(Cg)	18620	*Zdhhc11*^*tm1*.*1(KOMP)Vlcg*^/J	4	127	HET						
166	B6N(Cg)	24091	*Zfp346*^*tm1*.*1(KOMP)Vlcg*^/J	2	194/202	HET						X
	B6N(Cg)	24091	*Zfp346*^*tm1*.*1(KOMP)Vlcg*^/J	2	194/219	HET						X
167	B6N(Cg)	23023	*Zfp42*^*tm1b(KOMP)Mbp*^/J	2	151	HOM						
	B6N(Cg)	23023	*Zfp42*^*tm1b(KOMP)Mbp*^/J	2	145	HOM						
168	B6N(Cg)	24057	*Zfp961*^*tm1*.*1(KOMP)Mbp*^/J	2	179/226	HOM						
	B6N(Cg)	24057	*Zfp961*^*tm1*.*1(KOMP)Mbp*^/J	2	179/226	HOM						
169a	C57BL/6NJ	22366	*Zzef1*^*tm2(KOMP)Vlcg*^/J	2	198/206	HET						
	C57BL/6NJ	22366	*Zzef1*^*tm2(KOMP)Vlcg*^/J	2	269	HET						
169b	B6N(Cg)	22366	*Zzef1*^*tm2*.*1(KOMP)Vlcg*^/J	1	103	HOM						
	B6N(Cg)	22366	*Zzef1*^*tm2*.*1(KOMP)Vlcg*^/J	2	95/352	HOM						
	Females											
	Males											
	Strain not listed as B6N(Cg)											
HET	Heterozygous mutant mice											
HOM	Homozygous mutant mice											
	Normal skin and adnexa											
	Lesion in skin or adnexa											
X	Prenatal lethal or subviable phenotype											
	Clinically normal phenotype											

**Fig 1:**
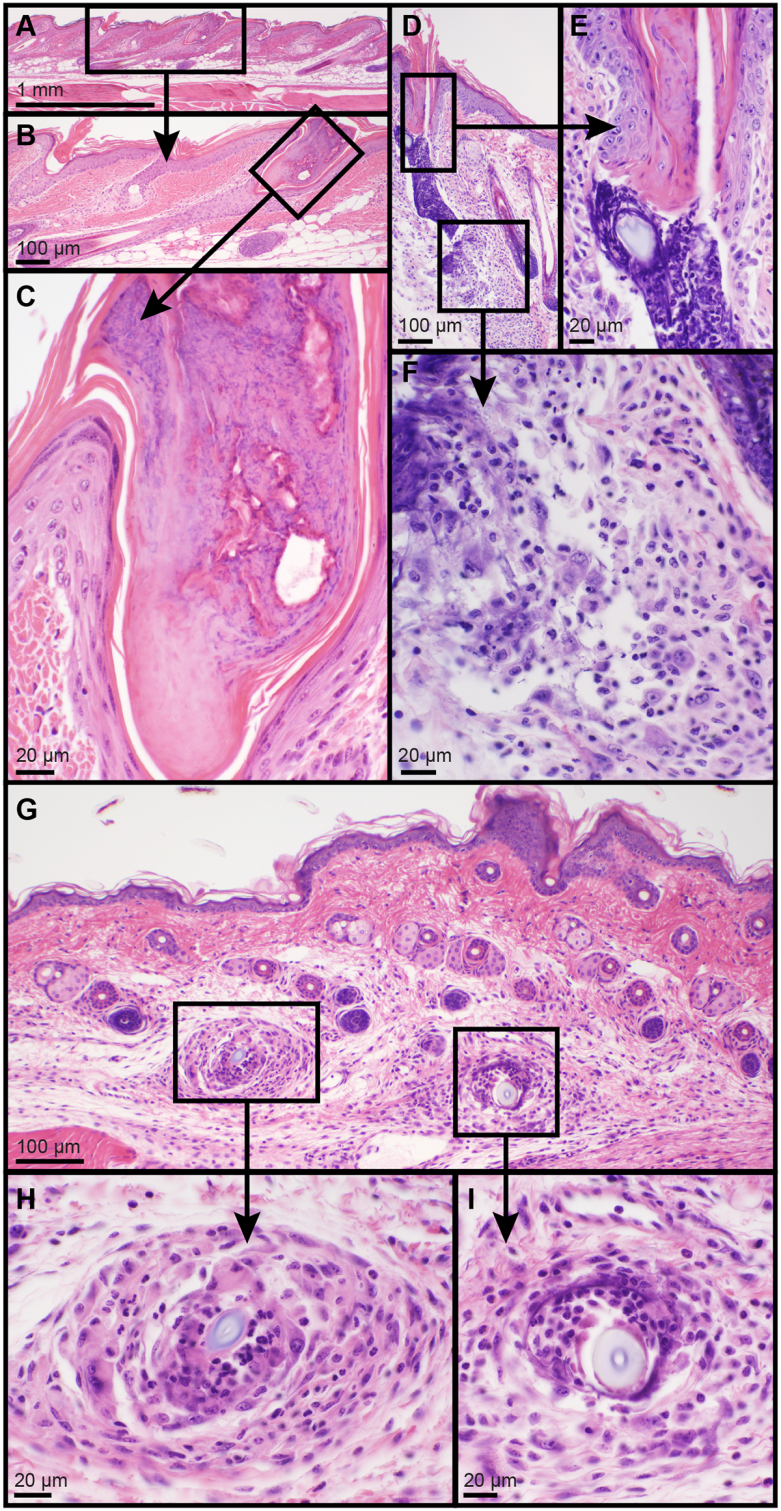
Incidental lesions were found scattered throughout the skin samples in many lines. Incidental lesions were not consistently found in all mice of a line. Lesions included solitary tail skin hair follicle with infundibular plugging (A-C). Truncal hair follicle infundibular plugging, rupture below the level of the sebaceous gland, follicle filled with neutrophils and surrounded by neutrophils and macrophages (D-F). Sporadic skin lesions found in many mice examined included isolated, one or two, ruptured hair follicles in the dermis where the free hair shaft fragments were engulfed by granulomas and surrounded by neutrophils (G-I). Hematoxylin and eosin stain, bar size indicated in each figure.

Nonspecific, sporadic lesions were found in many of the mice in this study. Most commonly these were ruptured hair follicles with hair shaft fragments free in the dermis or hypodermal fat layer surrounded by a variety of inflammatory cell types, usually neutrophils around the hair shaft and a layer of macrophages (trichogranuloma, Fig 1). These were often solitary and not consistently found in all the mice within a mutant line, which was a primary criterion to differentiate these lesions from gene mutation associated phenotypes. Other mice had follicular dystrophy consistent with C57BL/6 alopecia and dermatitis, a low frequency, strain-specific skin disease resembling human central centrifugal cicatricial alopecia [16].

**Fig 2:**
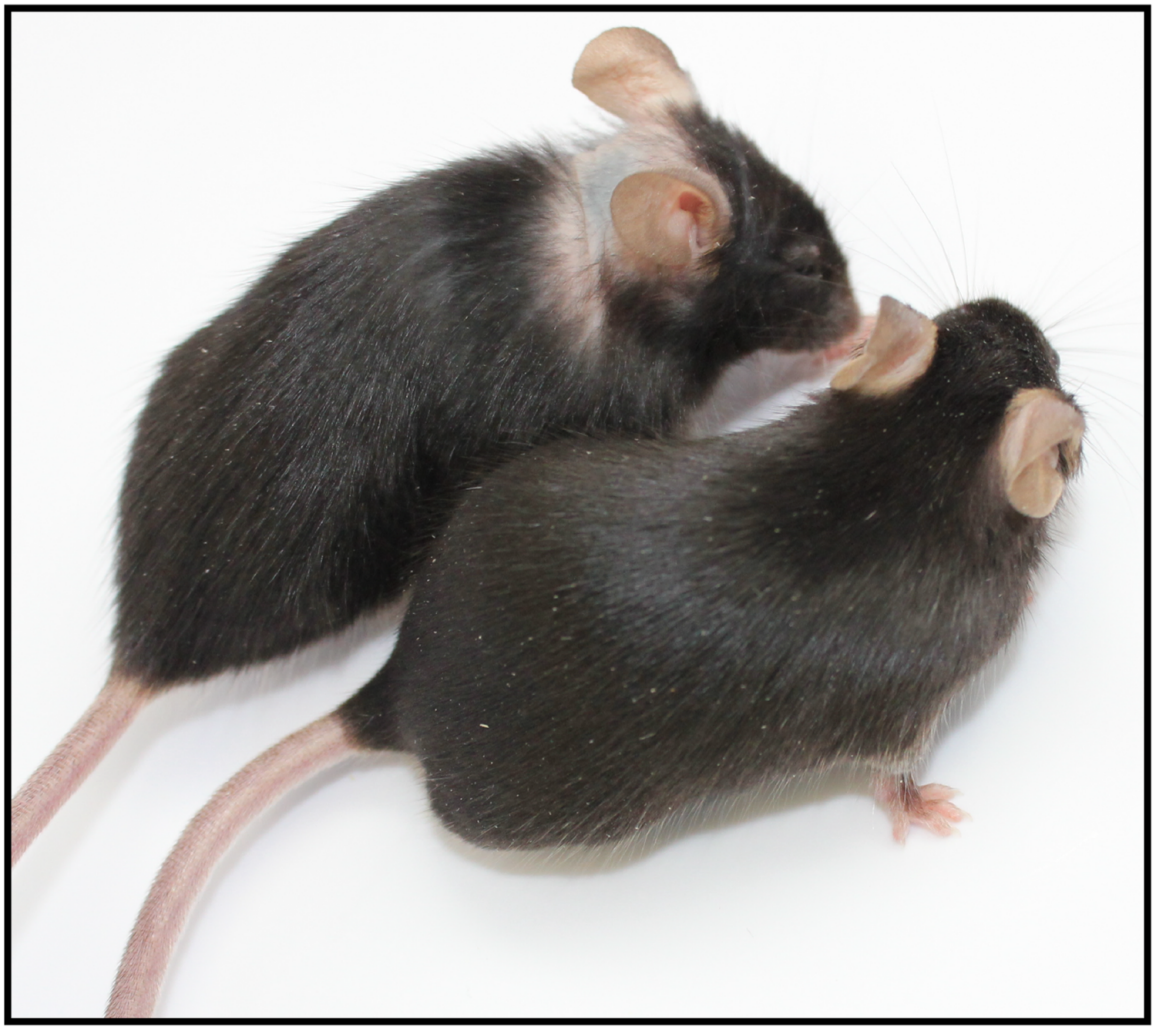
A B6N(Cg)-*Far2*^*tm2b(KOMP)Wtsi*^/2J mutant (left) and wildtype (right) mouse. Both are 15 week old females. Note the alopecia extending distally from the base of the ears.

**Fig 3:**
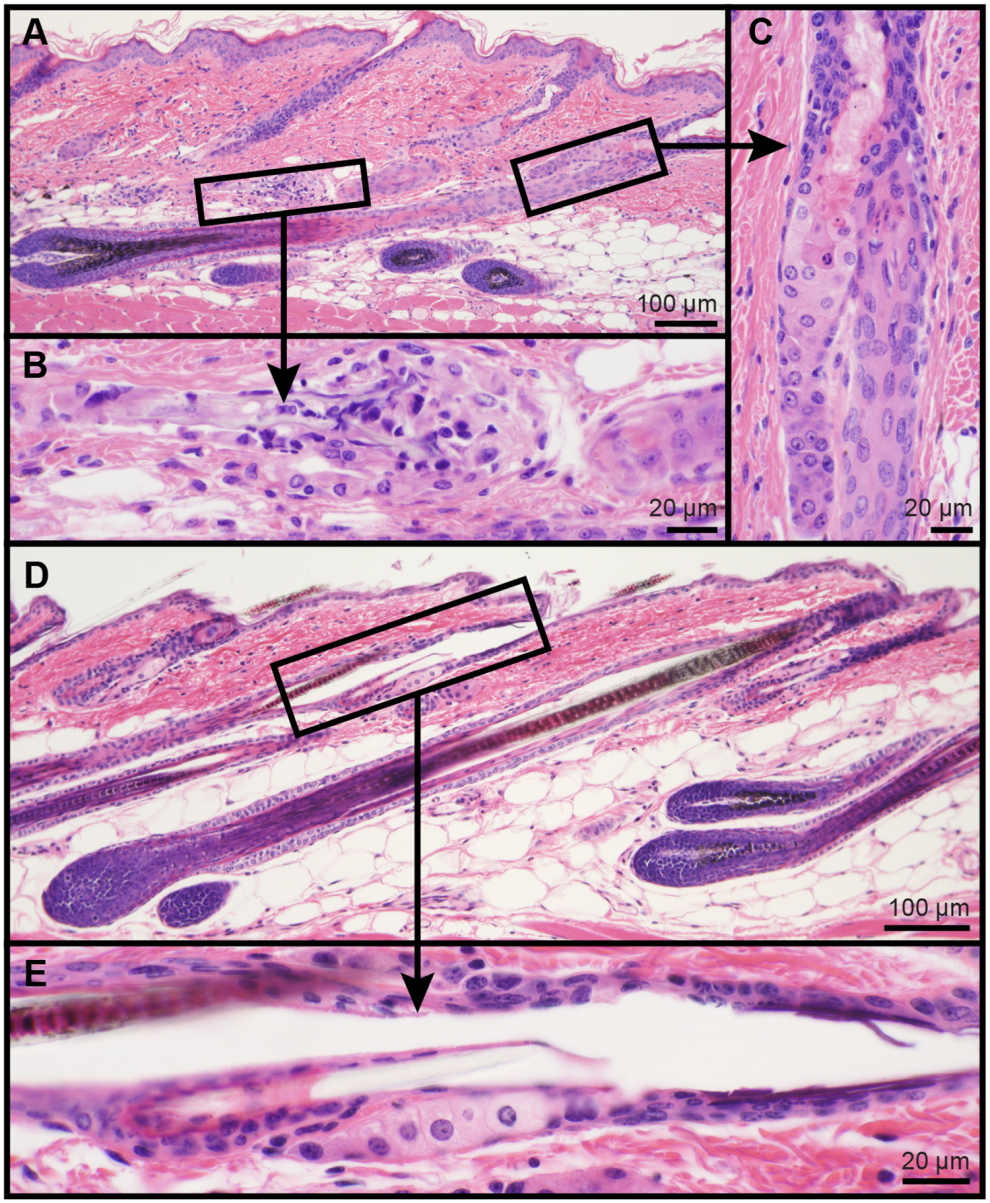
Sebaceous gland changes in fatty acyl CoA reductase 2 mutant mice. Most late stage anagen hair follicles in B6N(Cg)-*Far2*^*tm2b(KOMP)Wtsi*^/2J mutant mice, as in this 184 day old male, were relatively normal and produced a normal hair shaft (A). However, the sebaceous glands were mildly hypoplastic with brightly eosinophilic mature sebocytes that did not immediately rupture as they entered the infundibulum (C). As in other mouse mutations that cause hypoplasia of sebaceous glands, the hair shafts in these mice did not exit the follicular ostium resulting in perforation of the root sheaths, release of the hair shaft into the dermis, and an inflammatory reaction (B). This healed by follicular scarring, or cicatricial alopecia. By contrast, aged and sex matched B6N(Cg) controls rarely exhibited ruptured follicles (D). Sebaceous glands varied in size with the hair cycle, which was normal, and sebocytes had light pink cytoplasm that becomes very pale as the cells matured and abruptly ruptured into the duct. (E). Hematoxylin and eosin stain, bar size indicated in each figure.

**Fig 4:**
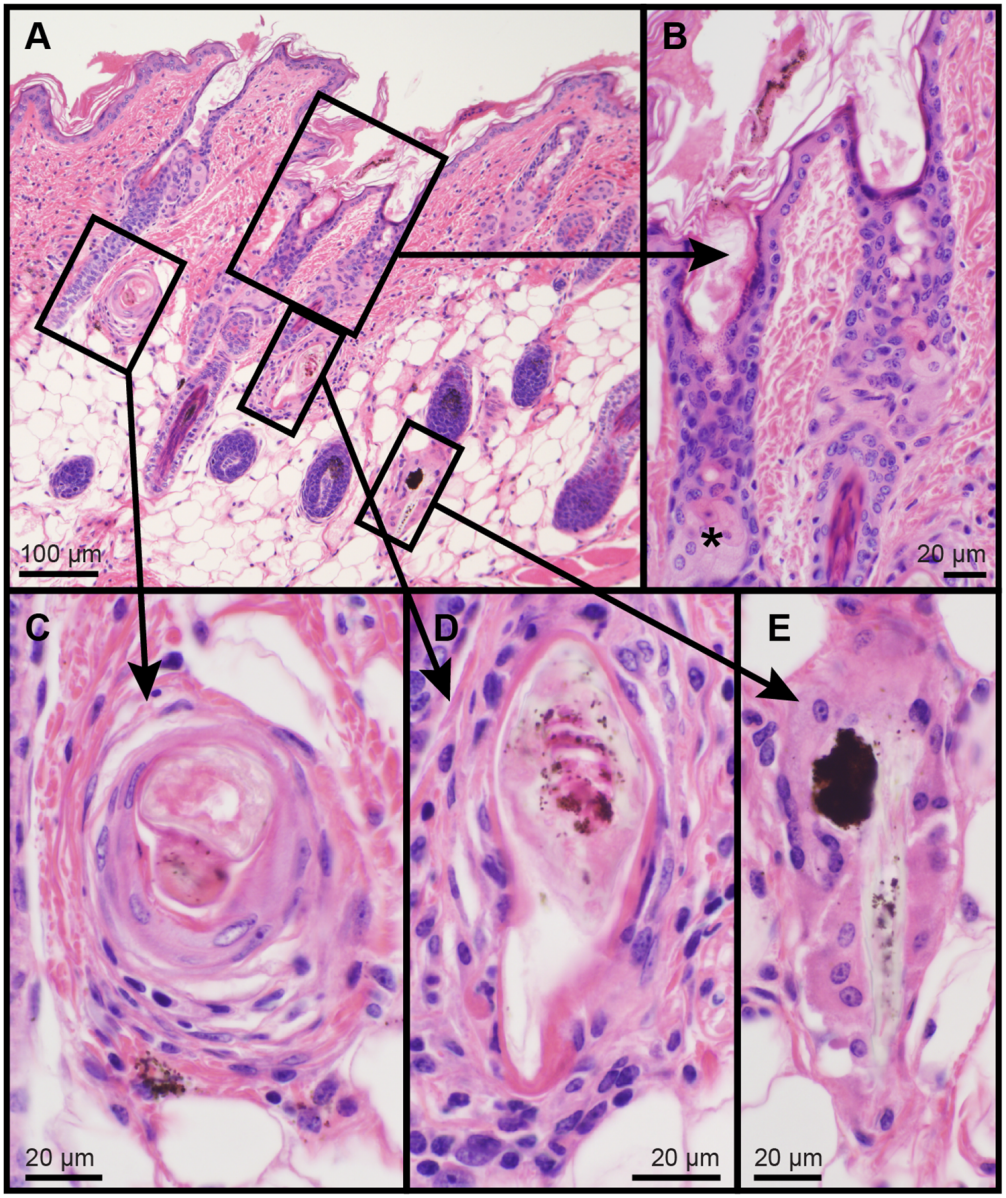
Follicular dystrophy in fatty acyl CoA reductase 2 mutant mice. In areas of alopecia, the B6N(Cg)- *Far2*^*tm2b(KOMP)Wtsi*^/2J mutant skin was mildly acanthotic and orthokeratotic, most likely secondary to the abnormal secretions of the sebaceous glands. The infundibulum was mildly dilated (A, B). Follicles with follicular dystrophy (C, D) and with pigment cast (E) were identified occasionally to frequently. Such ruptured follicles with granulomatous inflammation eventually lead to follicular scarring, a form of cicatricial alopecia. Hematoxylin and eosin stain, bar size indicated in each figure.

Mice with null mutations in their fatty acyl CoA reductase 2 (*Far2*^*tm2b(KOMP)Wtsi*^/2J) gene were found to consistently develop focal alopecia as adults on the dorsal skin extending distally from the base of the ears (Fig 2). Histologically, in areas where the skin appeared to be relatively normal there were minor lesions affecting the pilosebaceous and Meibomian (eyelids) glands that could be easily over-looked. Other modified sebaceous glands (Zymbal, clitoral, or preputial glands) were not examined in this study. The sebaceous glands superficially appeared to be normal but within the sebaceous duct the sebocytes became brightly eosinophilic and, when these holocrine cells ruptured, their secretion remained present and clumped. By contrast, normal sebocytes were slightly basophilic with fine, uniformly sized vacuoles in their cytoplasm. When the normal cells ruptured there was no evidence of their cytoplasmic content within the follicular infundibulum or on the skin surface (Fig 3). In areas of alopecia, the epidermis was mildly acanthotic with orthokeratotic hyperkeratosis associated with areas of follicular dystrophy and rupture and an associated foreign body inflammatory response that would lead to follicular scarring, a form of cicatricial alopecia (Fig 4). Skin surface lipidomic and time course studies are in progress to better define these changes. Further, non-integument phenotypes affecting lipid metabolism, behavior, immunity, and the cardiovascular system have been reported for this strain, and can be found on the IMPC database website (http://www.mousephenotype.org/data/genes/MGI:2687035#section-associations).

**Fig 5:**
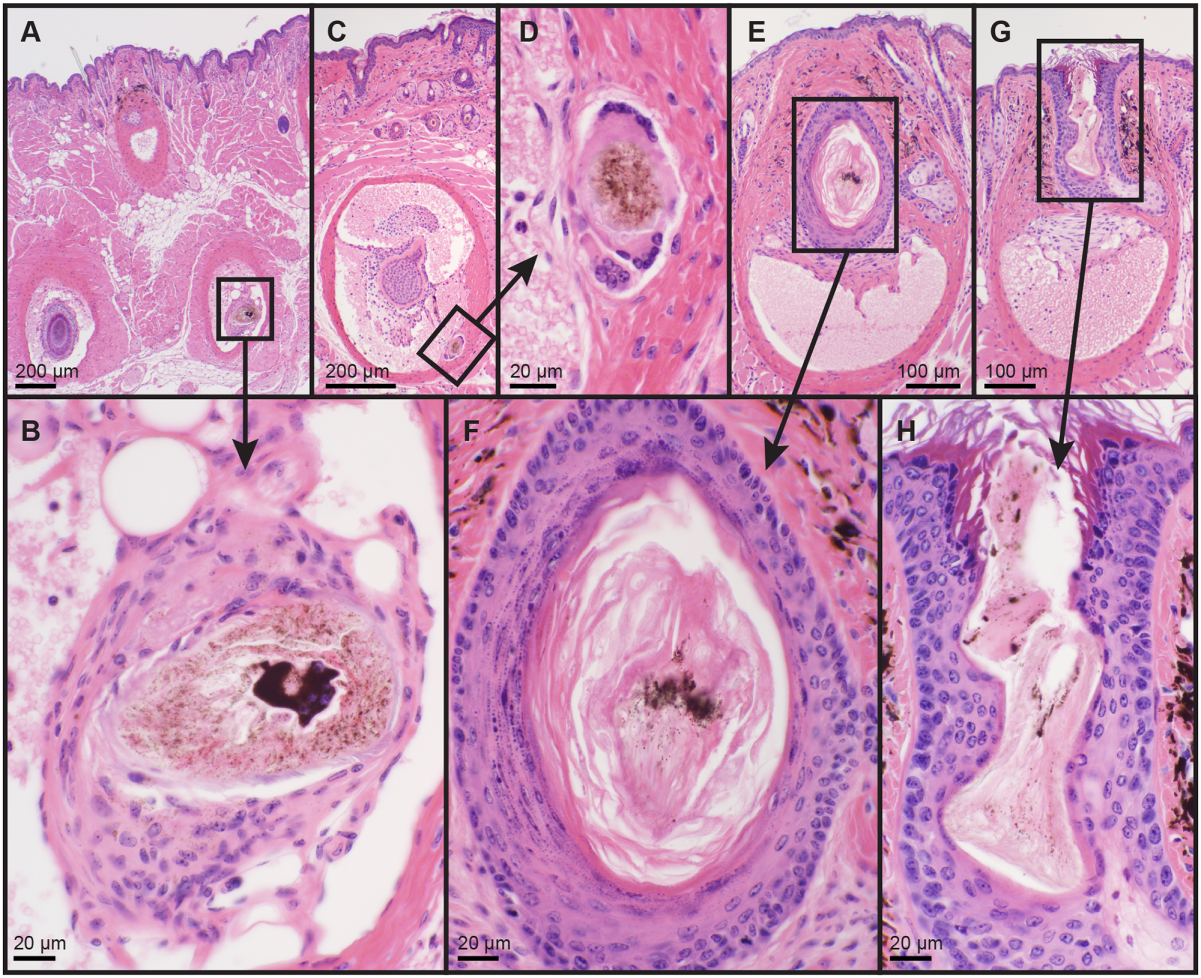
Vibrissae dystrophy in some protein phosphatase 1, regulatory subunit 9B mice. B6N(Cg)-*Ppp1r9b*^*tm1*.*1(KOMP)Vlcg*^/J, had follicular dystrophy limited to many but not all mystacial vibrissae in heterozygous but not homozygous mutant mice suggesting this is an incidental, albeit relatively frequent lesion. Boxed areas are enlarged as indicated by the arrows. Pigment casts are identified in all dystrophic hair follicles (B, F, and H). Hematoxylin and eosin stain, bar size indicated in each figure.

Heterozygous mutant mice for the protein phosphatase 1, regulatory subunit 9B (*Ppp1r9b*^*tm1*.*1(KOMP)Vlcg*^/J) gene had between one and five vibrissae hair shafts undergoing degenerative changes (follicular dystrophy) in each of three females and two males examined. Each section contained 10–15 vibrissae follicles (Fig 5). Only mystacial vibrissae were affected, not the periorbital or lower leg vibrissae. No other hair types were affected. This change was not found in two female and two male homozygous mutants suggesting that this was a nonspecific background lesion, as vibrissae follicular dystrophy was also seen occasionally in many mouse lines but only in one or two follicles and not consistently in all vibrissae in any section.

The skin and nail sections from almost all of the mice in these studies were clinically and histologically normal.

## Discussion

We have investigated the apparent deficiency of skin and adnexal phenotypes in a random set of strains from a systematic, genome-wide, knockout study using histopathological phenotyping. We report here that numbers detected confirm an earlier study [14] which also showed low numbers of detectable skin phenotypes, suggesting that ascertainment is not the cause of the “missing phenotypes”.

Of over 100 homozygous mutants screened, only one line, *Far2*^*tm2b(KOMP)Wtsi*^/2J, had lesions affecting sebaceous glands with what appeared to be a secondary follicular dystrophy, which probably progresses to a form of cicatricial alopecia as seen with a number of other mouse mutations with abnormalities of the sebaceous glands [17]. A rare autosomal recessive neurological disease without hair abnormalities was reported for humans with mutations in *FAR1* [18]. While the biochemistry of these two proteins, FAR1 and FAR2, is known no disease is currently associated with mutations in *FAR2* in humans (http://omim.org/entry/616156?search=Far2&highlight=far2). However, *Far2* transcripts were reported in the eyelid and skin, presumably localized in the Meibomian and sebaceous glands, respectively [19]. Information obtained from phenotyping these mice will be useful to help assess sebaceous gland abnormalities in humans as not all genes that are expressed in sebaceous glands result in sebaceous gland abnormalities or clinically obvious hair problems, such as mouse null mutations in *Soat1* [20, 21]. Detailed studies are in progress to define that pathogenesis of the follicular dystrophy and to match it with a specific human disease in these *Far2* mutant mice.

A second line, *Ppp1r9b*^*tm1*.*1(KOMP)Vlcg*^/J, had follicular dystrophy limited to many but not all vibrissae in heterozygous but not homozygous mutant mice suggesting this was a nonspecific background problem. A targeted mutant mouse line made for this gene (*Ppp1r9b*^*tm1Jfe*^) was reported to only have neurological abnormalities [22, 23]. As the vibrissae are an important somatosensory organ in the mouse [24] this structure should be carefully evaluated in such phenotyping studies.

The International Mouse Phenotyping Consortium has provided a unique, internally consistent dataset with genome-wide coverage and has yielded a great deal of valuable information on gene function. At the outset anticipation was high that systematic creation of null mice for all known protein coding genes would yield large amounts of information on the pathobiology of human disease. The high frequency of embryonic and neonatal lethal lines [4], consistent with previous estimates [6], was not a surprise, but potentially limits the value of these mice for detailed investigation of adult phenotypes and diseases which will require carefully designed conditional strategies.

The relatively low frequency of skin phenotypes in the International Mouse Phenotyping Consortium mice found in this and an earlier study [11] was surprising given the frequency expected from global surveys of MGI and OMIM discussed above. The histopathological survey presented here was very detailed focusing on the skin, and we believe this supports the assertion that the “missing phenotypes” are not due to lack of ascertainment, at least in the random sample selected. The remaining possibilities are that genes involved in skin morphogenesis and physiology are overrepresented in those essential for embryogenesis, the C57BL6N background is failing to show phenotypes, or that the complete removal of gene function in the mouse knockouts have more severe functional consequences than the point mutations, regulatory lesions, and insertions/deletions (indels) that constitute most disease-causing mutations in humans. Protein truncating variants which provide the greatest likelihood of complete functional loss (“knockouts”) are very rare in the human population [25].

In the current study, 74% of the heterozygous lines were pre-wean lethal or sub-viable as homozygotes (that were not examined) presenting the possibility that mutants having skin phenotypes might also be embryonic essential genes. While it is possible to argue that essential genes should never show up as disease genes in a population due to negative selection, there is increasing evidence that essential genes are often ‘disease genes”–i.e. carry mutations that are associated with abnormal or disease phenotypes. This is by no means a settled conclusion however. The relationship between gene essentiality and disease association is discussed in detail in Cooper et al. [25].

Studies conducted around a decade ago, based on a limited gene set, suggested that mouse genes that are essential—i.e., genes related to more severe phenotypes in mouse, are more likely to be human disease genes, but that embryonic lethals were less likely [26]. However, a more recent study strongly suggested that about 30% of human disease genes (from OMIM) have a mouse ortholog with a lethal knockout phenotype (673/1965) [27]. The discrepancies here reflect to an extent the methodology used to calculate the association and the inclusion or exclusion of genes with no phenotypic data in the study by Park *et al*. [26]. This association will certainly stand further scrutiny now that more data are available, but does not explain why the IMPC/KOMP strains show fewer skin phenotypes than the bulk of the loss-of-function allele carrying strains in MGI. Core analysis carried out on Ingenuity^™^ using the lists of lethal essential genes from supplementary table 3 of Dickinson *et al*. [4] shows no significant enrichment of annotations to dermatological disease or skin development, mitigating against the possibility that genes associated with skin disease are overrepresented in the essential segment of the genome. A working conclusion, therefore, is that either there is a problem with the strain background in this resource showing full expressivity of mutations affecting the skin, or that we have so far insufficient coverage of the genome using high-resolution phenotyping to account for the “missing phenotypes” from the International Mouse Phenotyping Consortium program. Of concern is the observation that most human disease associated mutations are hypomorphic alleles which, in the event of lethal homozygosity of complete KOs, are much more likely to present a phenotype compatible with survival. The potential for missing these with a complete loss of function strategy is significant, especially when examining completely novel genes where there are no existing experimental or inferred annotations. The power of looking for skin phenotypes using a strategy such as that developed by Beutler *et al*. [28] using ENU mutagenesis is very persuasive. Currently Mutagenetix (https://mutagenetix.utsouthwestern.edu/home.cfm), accessed 1 Mar 2017) shows 49 genes with skin associated phenotypes recovered as phenotypic mutations from a total of 262 phenotyped genes (119 of 498 mapped mutations) and should be considered in the future along with full characterization of spontaneous and ENU generated mutations in large collections.

## Materials and methods

### Generation of mutant mice

#### Embryonic stem cell lines

Initially mice included in the program were produced from embryonic stem cell lines acquired from repositories (https://www.komp.org/ or http://www.eummcr.org/). These resources comprised two major allele types, the Velocigene “definitive null” allele produced by the Regeneron Corporation (*Vlcg*) [1] and the “knockout first, conditional ready” design produced by the Wellcome Trust Sanger Centre and partners (Sanger) [2]. The *Vlcg* alleles delete the entire coding sequence of the target gene, including all introns, while inserting a *LacZ* reporter cassette. The *neo* resistance cassette is flanked by *loxP* sites for removal by *cre*-recombinase. The Sanger allele is a multifunctional design. In its native form, the strong splice acceptor typically generates a null allele. When bred to *Flp*-expressing mice, the entire 5’ *LacZ* and *neo* cassette are removed leaving a *loxP*-flanked critical exon for conditional mutagenesis. When bred to *cre*-recombinase transgenic mice, this allele is converted into a “definitive null” which retains the 5’ *LacZ* cassette and removes the critical exon and *neo* marker.

All animal work was approved by The Jackson Laboratory Animal Care and Use Committee (approval number 07005 "Mouse Models for Dermatology Research”).

#### Generating live mice

Following importation at The Jackson Laboratory, clones are injected into blastocyst hosts and resulting chimeras are bred to C57BL/6NJ (STOCK#5304) or C57BL/6NJ-*Tyr*^*em3J*^/GrsrJ (STOCK#21999; to select for coat color transmission) inbred mice (see Table 1) to assure isogenicity of the resource. Germline transmitting (GLT) clones are quality confirmed (both *Vlcg* and Sanger) for targeting. Following GLT, mice are bred to congenic *cre*-recombinase transgenic mice (B6N.Cg-Tg(*Sox2-cre*)1Amc/J; STOCK#14094) to remove the *neo* cassette and to generate a definitive null allele, generating the tm1.1 allele (*Vlcg* and non-conditional Sanger clones) and *tm1b* allele (from conditional-ready Sanger clones). Four lines were generated by CRISPR/Cas9 mutagenesis, using standard approaches [29] to generate small deletions in the early part of the transcript. These mice are expanded, quality control-confirmed, and intercrossed to generate knockout mice. Approximately 30% of lines display lethality or sub-lethality (defined as yielding less than 12.5% homozygous mice) [4]. For these strains, heterozygous mice were phenotyped. For other strains, homozygous breeder pairs established for colony maintenance used in this study when they became retired breeders to maximize chances of finding adult onset diseases.

### Systematic analysis of mutant mice

#### Animal logistics

Two female and two male retired breeder mice were provided from The Jackson Laboratory knockout mouse production program (Table 1). Homozygous lethal embryos and perinatal lethal mouse lines were specifically excluded from these studies. In these cases, heterozygous animals were aged and evaluated. All lines, whether *Vlcg* definitive null or Sanger conditional-ready mice were evaluated post-*cre* excision, which removed the *neo* cassette and heterologous promoter and in the case of Sanger alleles also removing the critical exon to create a definitive null allele (*Vlcg tm1*.*1* and Sanger *tm1b*). All mice had skin, hair, and nails collected (see below).

#### Necropsy procedures, slide generation, and histopathologic analyses

As the skin is anatomically and molecularly distinct throughout the body in both mice and humans [30, 31] and diseases can be restricted to specific anatomic sites, skin was collected from several areas. As dorsal and ventral trunk skin are histologically similar in the mouse, they were collected with other anatomically identifiable tissues and put into respective cassettes: 1) dorsal skin and ear skin; 2) ventral skin, eyelid skin, muzzle skin; 3) digits to include the foot pads and nail unit; and 4) cross and longitudinal sections of the tail. This allows for evaluation of all major hair follicle types, mucocutaneous junctions, the nail unit, as well as the footpad (including eccrine glands) [32]. Detailed protocols on skin removal, orientation, and processing are described elsewhere [33]. Tissues were fixed in Fekete’s acid-alcohol-formalin solution. Sections were stained with hematoxylin and eosin (H&E) for routine screening.

## Abbreviations

Far2: fatty acyl CoA reductase 2 gene
GLT: germ line transmitting
H&E: hematoxylin and eosin stain
MGI: Mouse Genome Informatics Database
